# Supplementary material for: Optimized CRISPR tools and site-directed transgenesis towards gene drive development in Culex quinquefasciatus mosquitoes
Source: Nat Commun. 2021 May 20;12:2960. doi: 10.1038/s41467-021-23239-0 (PMC8137705; doi:10.1038/s41467-021-23239-0)
Supplement: Supplementary file 1 — Supplementary Information [file 41467_2021_23239_MOESM1_ESM.pdf]

Optimized CRISPR tools and site-directed transgenesis towards gene drive development in *Culex quinquefasciatus* mosquitoes.

Xuechun Feng[1], Víctor López Del Amo[1], Enzo Mameli[2,3], Megan Lee[1], Alena L. Bishop[1], Norbert Perrimon[2,4], Valentino M. Gantz[1].*

[1] Section of Cell and Developmental Biology, University of California San Diego, La Jolla, CA 92093, USA.

[2] Department of Genetics, Blavatnik Institute, Harvard Medical School, Boston, MA 02115, USA.

[3] Department of Microbiology, National Emerging Infectious Diseases Laboratories, Boston University School of Medicine, Boston, MA 02118, USA.

[4] HHMI, Harvard Medical School, Boston, MA 02115, USA.

*Correspondence: vgantz@ucsd.edu

Keywords: population engineering, insect control, mosquito, HDR, NHEJ, cardinal, Drosophila melanogaster, gRNA, scaffold.

SUPPLEMENTARY INFORMATION

Supplementary Figure 1

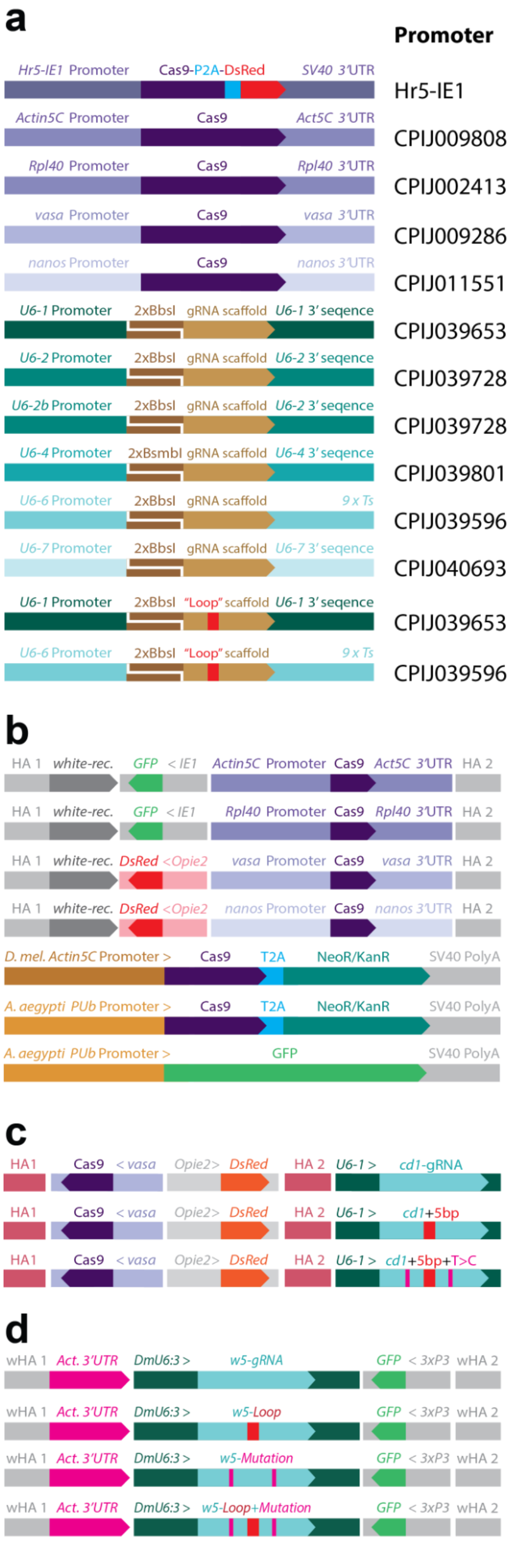

Promoter	Strain	Plasmid	Accession	Addgene ID
Hr5-IE1	n/a	pVMG225_IE1>Cas9-P2A-DsRed	-	-
CPIJ009808	CA	pVMG173_*Cq-Actin5c*>Cas9	MW925696	169345
CPIJ002413	CA	pVMG193_*Cq-Rpl40*>Cas9	MW925697	169346
CPIJ009286	CA	pVMG213_*Cq-vasa*>Cas9	MW925698	169347
CPIJ011551	CA	pVMG212_*Cq-nanos*>Cas9	MW925699	169348
CPIJ039653	AL	pVMG146_*Cq-U6:1*>2xBbsI-gRNA	MW925700	169238
CPIJ039728	AL	pVMG147_*Cq-U6:2*>2xBbsI-gRNA	MW925709	-
CPIJ039728	CA	pVMG217_*Cq-U6:2b*>2xBbsI-gRNA	MW925701	169339
CPIJ039801	CA	pVMG218_*Cq-U6:4*>2xBsmbI-gRNA	MW925702	-
CPIJ039596	AL	pVMG149_*Cq-U6:6*>2xBbsI-gRNA	MW925703	169323
CPIJ040693	CA	pVMG164_*Cq-U6:7*>2xBbsI-gRNA	MW925704	-
CPIJ039653	AL	pVMG302_*Cq-U6:1*>2xBbsI-gRNA-Loop	MW925707	169369
CPIJ039596	AL	pVMG303_*Cq-U6:6*>2xBbsI-gRNA-Loop	MW925708	169370
		pVMG177_*CqActin5c*>Cas9_wHAs	-	-
		pVMG220_*CqRpl40*>Cas9_wHAs	-	-
		pVMG236_*CqVasa*>Cas9_wHAs	-	-
		pVMG235_*CqNanos*>Cas9_wHAs	-	-
		p*Dm-Act5C*>Cas9-T2A-Neo	-	-
		p*Aae*-PUb>Cas9-T2A-Neo	-	-
		pSL1180-HDR-*Aae*-PUb>GFP-NLS	-	-
		pVMG252_*Cq-vasa*>Cas9_cdHAs_O	MW925705	-
		pVMG280_*Cq-vasa*>Cas9_cdHAs_L	-	-
		pVMG277_*Cq-vasa*>Cas9_cdHAs_L+M	-	-
		pVMG109_CC-*U6:3-w5*_GFP_wHAs_O	MW925706	-
		pVMG221_CC-*U6:3-w5*_GFP_wHAs_L	-	-
		pVMG222_CC-*U6:3-w5*_GFP_wHAs_M	-	-
		pVMG274_CC-*U6:3-w5*_GFP_wHAs_L+M	-	-

Supplementary Figure 1 - Reagents that have been built for the experiments in this manuscript. **(a)** Transgenes built for the expression of Cas9 and gRNA. The gRNAs constructs have been further

modified by adding the gRNA target sequences or the scaffold variants, such further edits are not reported in this table. Next to the construct structure are reported the genes from which genomic sequences were amplified and the laboratory line from which genomic DNA and PCR amplification was run. **(b)** Variation of the Cas9 constructs used to test activity in cells and in embryos. These constructs were tested instead of the simpler ones as we originally intended to target the white gene for transgenesis. Additionally we tested two exogenous promoters, *Actin5C* from *Drosophila melanogaster* (*D. mel.*) and *PUb* from *Aedes aegypti*. (**c**) HR template plasmids built for transgenesis of the vasa-Cas9 cassette in *Culex quinquefasciatus.* (**d**) Constructs used to evaluate transgenesis and gene drive performance in *Drosophila melanogaster.* Accession numbers for the deposited plasmids are also provided below and in the Data Availability Statement.

Supplementary Figure 2

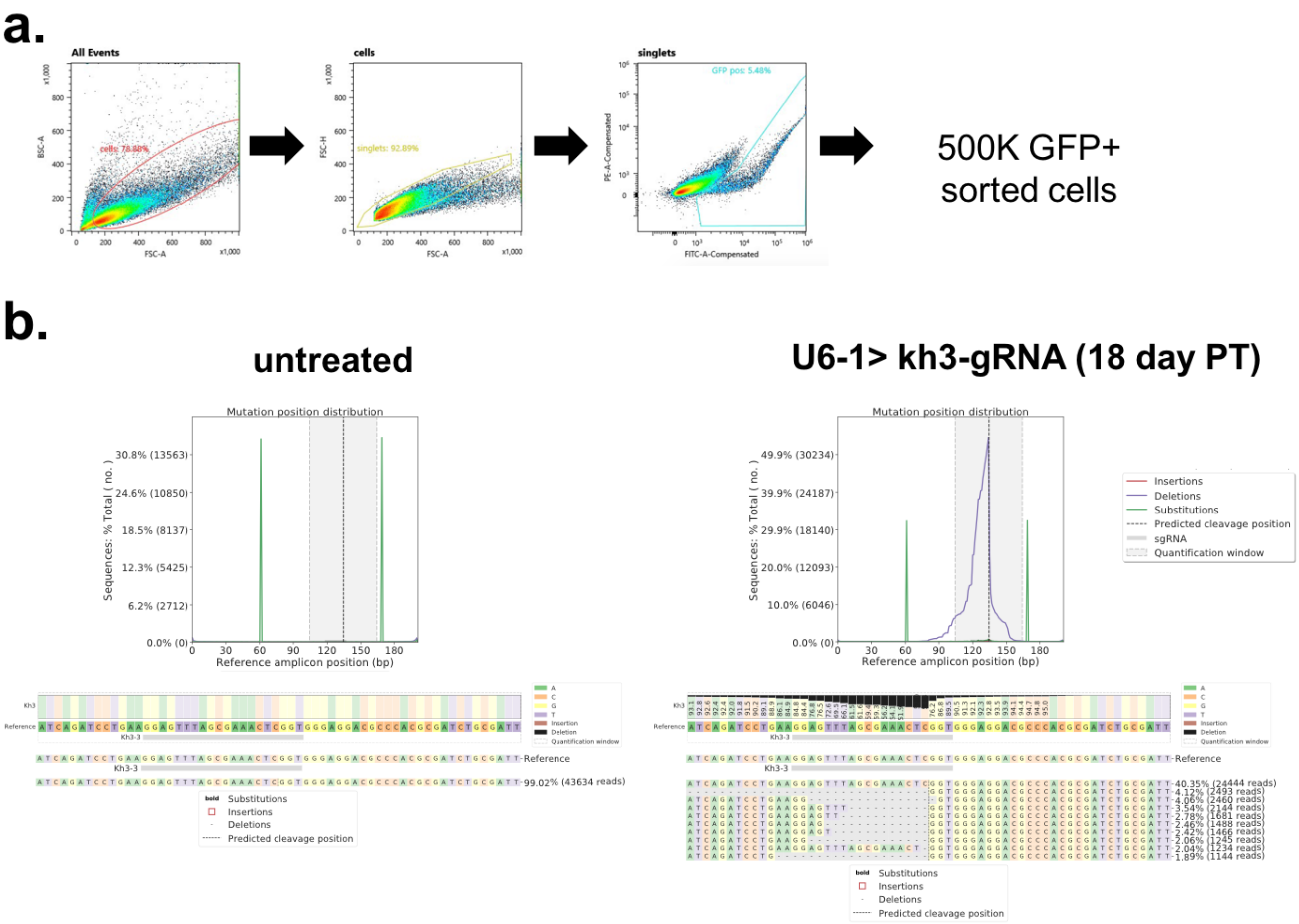

Supplementary Figure 2 - FACS gating strategy and CRISPResso2 analysis output. (a) Gating strategy used for FACS sorting of GFP expressing cells. **(b)** Example of CRISPResso2 analysis output for untreated and kh3-gRNA targeted sample. The graph shows all types of mutation occurring in the kh3 target amplicon represented by category; The quantification window is highlighted in gray and the broken line represents the exact cutting site (-3bp from PAM). Below is represented the % of nucleotide substitutions or deletions around the cutting site. At the bottom, a 60bp sequence window centered on the cutting site shows mutated allele sequences and their relative % abundance.

Supplementary Figure 3

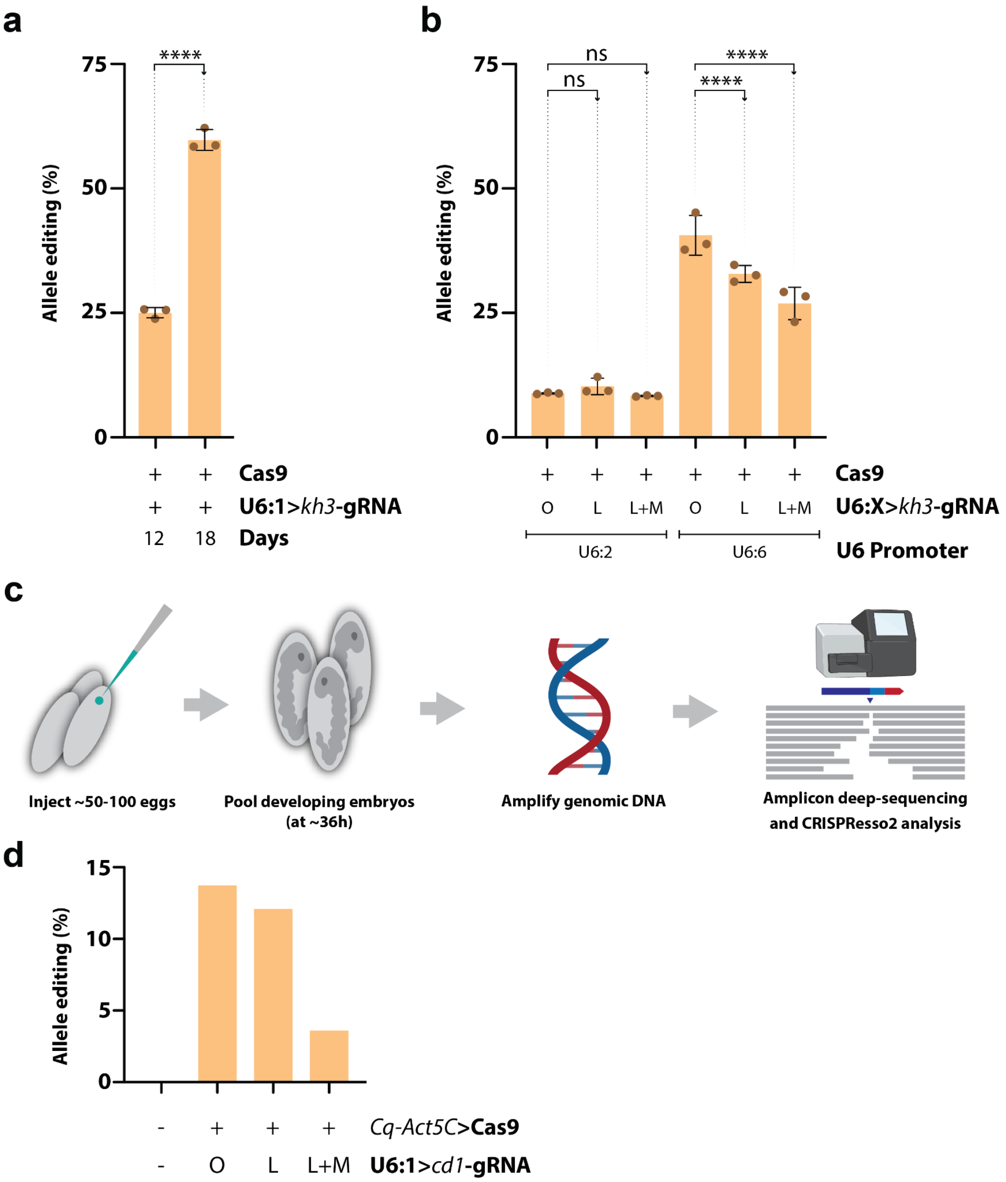

Supplementary Figure 3 - Additional testing of the *Culex quinquefasciatus* reagents in cells and *in vivo*. **(a)** Histogram representing editing efficiency (%) of *kh3* target locus in Hsu cells when co-transfected with a mixture of *Cq-Actin5C*>Cas9 paired to U6:1>*kh3*-gRNA and analyzed 12 days (left bar) or 18 days (right bar) post-transfection. **(b)** Editing efficiency (%) at *kh3* target locus in Hsu cells when transfected with a mixture of *Cq-Actin5C*>Cas9 paired either to U6:2 or U6:6 *pol-III* promoters expressing the *kh3*-gRNA with different gRNA scaffold variants. Histogram bars represent the mean, error bars and dots represent SD and distribution of 3 biological replicates. **(a-b)** Statistical comparisons were generated with a two way ANOVA multiple comparison of the mean, with Tukey's corrections. **** = $P_{tukey} < 0.0001$, ns = $P_{tukey} > 0.05$. P-values: **(a)** 12d-18d<0.0001; **(b)** U6-2_O-L=0.9997; U6-2_O-L+M>0.9999; U6-6_O-L<0.0001; U6-6_O-L+M<0.0001. **(c)** Modified version of our protocol in **Fig. 1d** that we used to evaluate *in vivo* the allele editing rates of the gRNA variants. The *Cq-Actin5C*>Cas9 plasmid was co-injected with U6:1>*cd1*-gRNA with gRNA scaffold variants, then we collected DNA from all developing embryos at ~36h post injection for the subsequent deep sequencing analysis. **(d)** Edited allele percentages observed at the *cd1* site, for the scaffold

variants evaluated. The control was generated from a pool of uninjected eggs. **(b, d)** O = "Original", L = "Loop", L+M = "Loop+Mutation". **(c)** Partially created with the help of BioRender.com.

Supplementary Figure 4

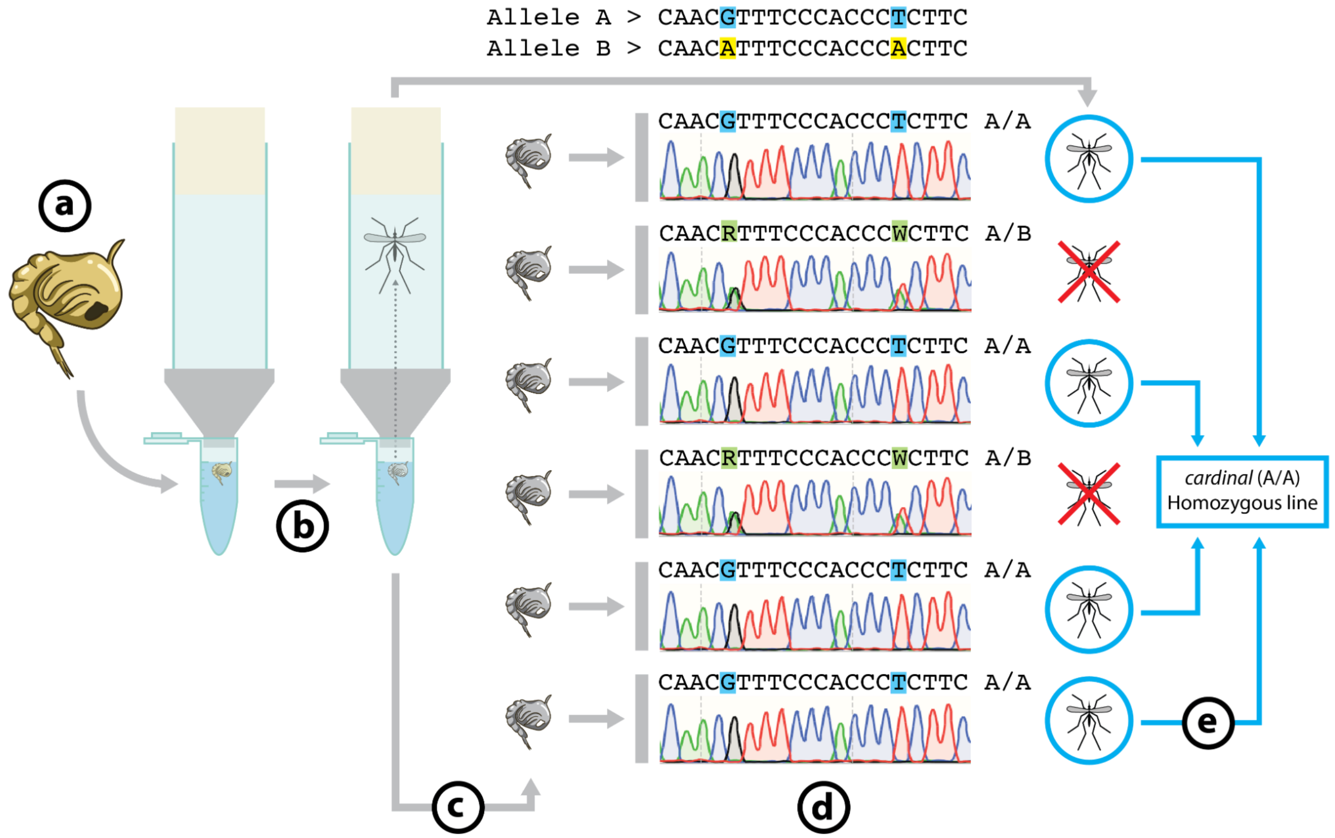

Supplementary Figure 4 - Generation of a *Culex quinquefasciatus* line isogenic at the *cardinal* locus. (a) Pupae about to hatch were collected and individually deposited in different Eppendorf tubes with about 1mL of water. Each tube was then coupled with a capped plastic *Drosophila* vial with the bottom sawed off, using a 3D-printed adaptor. **(b)** After ~24 hours the hatched mosquitoes were maintained in the respective tubes, while **(c)** the pupal case was removed and used for PCR amplification of the *cardinal* locus. **(d)** Resulting amplicons were used for Sanger sequence analysis of the *cardinal* locus and evaluate the individuals' genotype (A/A, A/B or B/B; we did not recover any B/B animals suggesting lethality associated with this allele). **(e)** All A/A male and female mosquitoes were pooled in a cage to generate a line homozygous for the cardinal-A allele, about ~24h after hatching. Note that the mosquitoes were able to survive overnight within the collection vials without needing a source of food or water as they were kept in a high-humidity environment.

Supplementary Figure 5

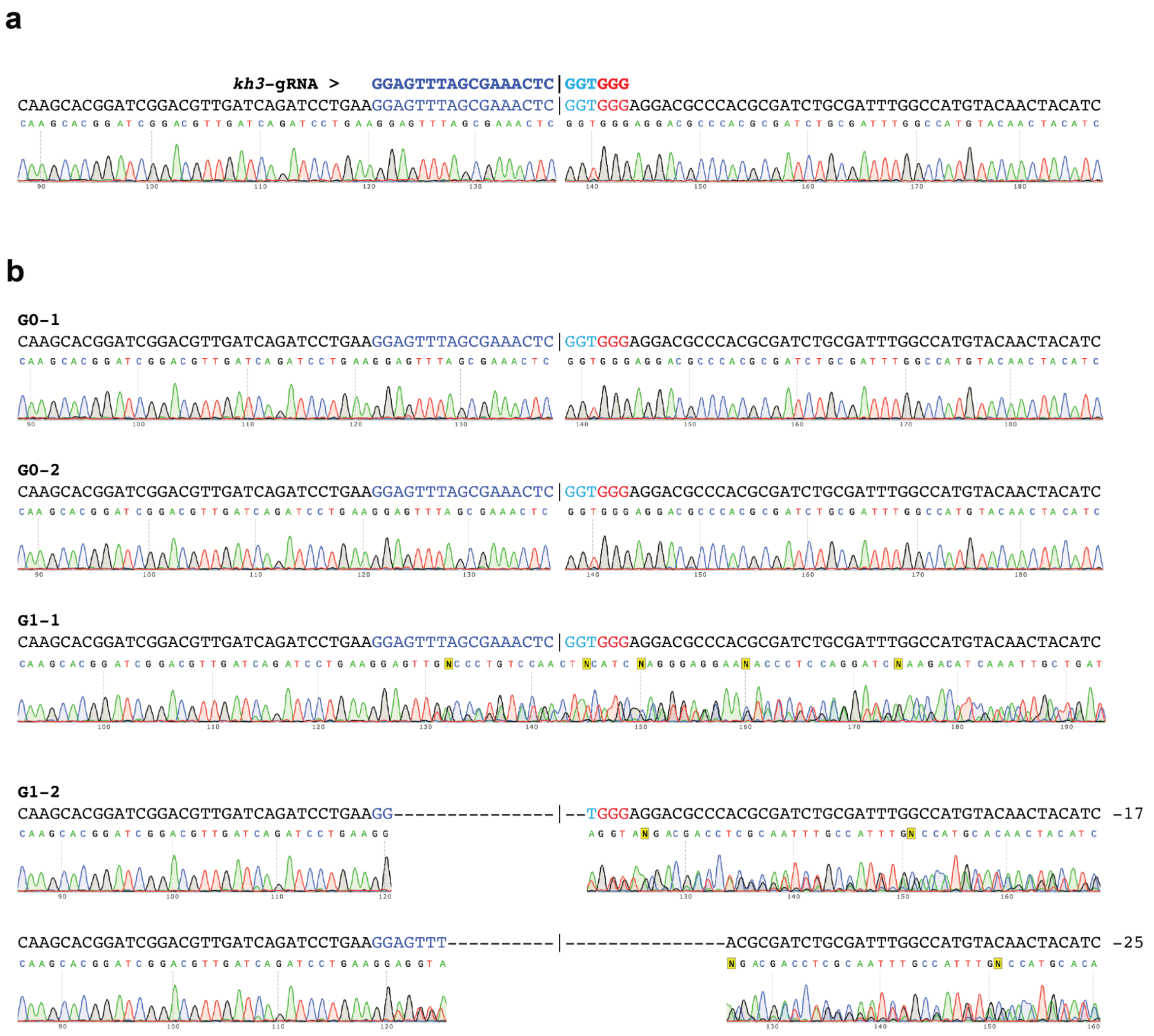

Supplementary Figure 5 - Sequencing of G0 and G1 animals from the Cas9-line validation experiment. (a) Wild-type locus, showing the location of the gRNA target in blue and the PAM in red. A vertical bar indicates the location of the cut site. **(b)** Sanger sequences traces obtained from two G0 injected mosquitoes showing no apparent cutting; and two traces obtained from two G1 animals displaying a *kh-* (white eye) phenotype. For the bottom animal (G1-2) we were able to disentangle the two indels present by using the ICE analysis software by Synthego (Synthego Performance Analysis, ICE Analysis. 2019. v2.0. Synthego; 15 Oct. 2020), with a Sanger sequencing read performed on wild-type as a control; we have aligned the same sequence trace at the positions corresponding with each indel.

Supplementary Figure 6

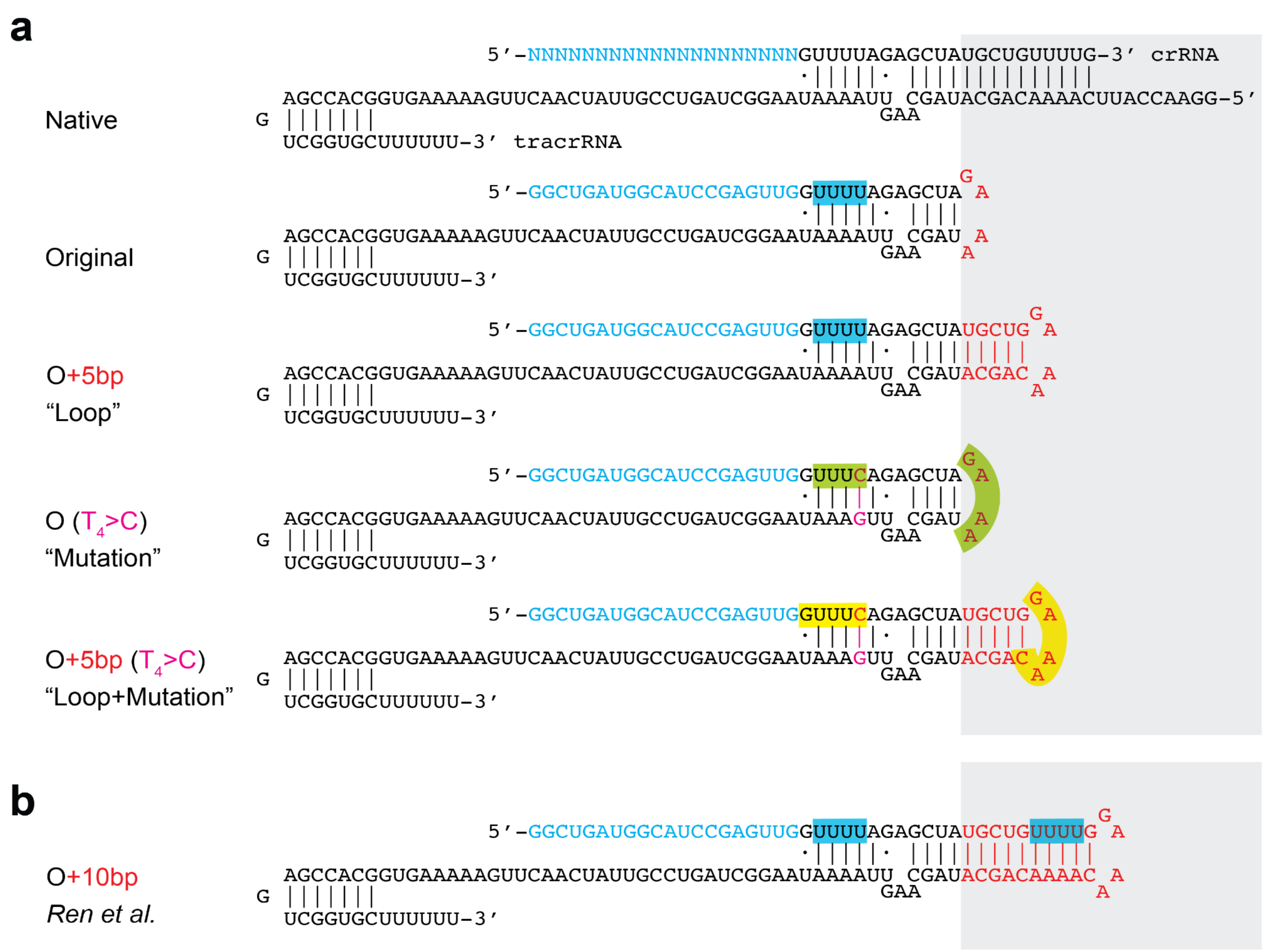

Supplementary Figure 6 - Potential mispairing of the scaffold variants containing the T_4>C mutation. (**a**) Shows the different gRNA variants tested in this study and their RNA base-pairing compared to the native crRNA/tracrRNA couple. For the gRNAs containing the T_4>C mutation, we identified a potential pairing between the sequence present in the first loop "GAAA" with a sequence present at the beginning of the gRNA scaffold UUUC, highlighted in green. When the mutation is combined with the loop, the added sequence introduces an even longer potential base-pairing of the GAAAC with the GUUUC sequence, highlighted in yellow. (**b**) Modified scaffold tested in *Ren et al.* with an additional 10bp in the loop. Highlighted in blue are the sequences, present as Ts in the transgene that could potentially trigger premature stop of transcription. The grey shaded areas in the figure highlight the synthetic portions of the gRNA variants that were introduced to link the crRNA and the tracrRNA.

Supplementary Tables

Supplementary Table 1 - Additional injection conditions with supplemented Cas9 protein and/or plasmid mixture.

Plasmid	Round of Injection	Cas9 Source		Injected G0			Efficiency (per G0 germline)*		Overall efficiency (out of total G1s)#	
		Protein	Plasmid Mix	Injected eggs	Adult survivors	Survival (%)	Cutting *cd-/cd-* (%)	Transgenic DsRed+ (%)	Cutting *cd-/cd-* (%)	Transgenic DsRed+ (%)
***cd1*-original**	1	+	+	320	36	11.25%	0/36 (0.00%)	0/36 (0.00%)	0/398 (0.00%)	0/398 (0.00%)
***cd1*-loop**	1	+^	+	460	9	1.96%	**3**/9 (33.33%)	**2**/9 (22.22%)	**48**/431 (11.14%)	**39**/431 (9.05%)
	2	+	+	412	16	3.88%	**1**/16 (6.25%)	0/16 (0.00%)	**15**/804 (0.19%)	0/804 (0.00%)
	1	+	-	280	13	4.64%	0/13 (0.00%)	0/13 (0.00%)	0/982 (0.00%)	0/982 (0.00%)
	1	-	+	550	55	10.00%	0/55 (0.00%)	0/55 (0.00%)	0/2666 (0.00%)	0/2666 (0.00%)

* The G0 germline cutting and transgenesis efficiencies were calculated as numbers of independent pools that produce either *cd-/cd-* mutant (cutting) or DsRed+ (transgenesis) animals, divided by the total number of crossed G0s. While each pool contains several G0 individuals, in our calculations we assume only one editing event happens in each positive pool, therefore our calculations might underestimate the cutting and transgenesis rates. # The overall cutting and transgenesis efficiencies were calculated as the number of G1 individuals with either *cd-/cd-* (cutting) or DsRed+ (transgenesis) phenotypes divided by the total number of G1s. ^ In this injection we used an older batch of Cas9 protein which later showed low activity in subsequent studies.

Supplementary Table 2 - Primer List

Name	Primer
**Primers used for building constructs**	
494	GGTATCAGCTCACTCAAAGGCGGTAATACGG
495	GAAAGGGCCTCGTGATACGCCTATTTTTATAGG
V0915	AATAGGCGTATCACGAGGCCCTTTCGACTAACGTACCAATGCACCTGG
V0889	GAAGACAGGCGAAAGCCAAACGTTTACGCTCACCTCCCGACAGACCCTTGATCCGTCCTG
V0888	CGTTTGGCTTTCGCctGTCTTCatctaGAAGACatCCGAGAGTGCGTCGTTCATCAAATAGGCGATCGCGGTGGGGAGAGGAAACGTCTG
V0914	GCCTTTGAGTGAGCTGATACCATTGCAAGTTCCTAACCATAACCTAC
V0700	GGCCAATTTGCCAATTTACCCCCG
V0638	CTATTTGATGAACGACGCACTCTCGG
V0882	CGAAGAAGAAGCGCAAGGTGTAATAAGCGCCTTCACGCCGCAACAAC
V0883	TCCAGACCGATCGAGTACTTCTTATCCATGTTGATATCTGCGCGCAAG
V0325	GATAAGAAGTACTCGATCGGTCTGGATATC
V0884	TTACACCTTGCGCTTCTTCTTCG
V0885	CGAGAGTGCGTCGTTCATCAAATAGGCGATCGCgGAGACCGCTCACTCAAAGGCGGTAATACGG
941	GGGCGAATTCTGCAGATATCCATCAC
V0887	GGTAAATTGGCAAATTGGCCGCGATCGCttaGGCGCGCCcgcgtaaaacacaatcaagtatg
V0886	GATGGATATCTGCAGAATTCGCCCTTGGTCTCaGGCGCGCCGGTCGACTGATCATAATCAGC
V0834	GCATTTTCTGCCTGCTTCTTTTCTCACGG
V0832	GTCATCGTGGTAAGCGAATCTAG
V0963	CCGAAGAAGAAGCGCAAGGTGTAAGcCCCCATTCGTTCGCCGTTTG
V0964	CCGATCGAGTACTTCTTATCCATGGTGAATCTGCGGGAACGGAAAG
V1044	GTTTCCTCTCCCCACCGCGATCGCGTCATCGTGGTAAGCGAATCTAG
V1040	CGCGGGCGCGCCtaaGCGATCGCGCATTTTCTGCCTGCTTCTTTTC
V0512	GCATACAGAGCTTAGCTCTGTTC
V0408	GATCGAATGCAATAATCACCACGTAAG
V1068	GATCGCGGTGGGGAGAGGAAACGTCTGG
V1069	GAAAGCCAAACGTTTACGCTCACCTCCCGACAGACCCTTGATCCGTCCtG
V1000	GTGAGCGTAAACGTTTGGCTTTC
V1070	atcgcttaGGCGCGCCcgcg
V0660	ATGTCCTTTTTAATCACGATTCAC
V0678	GGCTTTTTTACGAAAACCTCCAACTC
V1009	GACCGATCGAGTACTTCTTATCCATGTTTTAGAAAGGACCTTGcCGGG
V1011	ACAAATTTTGAGCAACCTTGCTCAAGGGCGAATTCTGCAGATATCC
V0982	AATTGTAGTAATTTGAACCATGTAAGAG
V0656	GAGCAAGGTTGCTCAAAATTTGTCTG
V1010	GAAGAAGAAGCGCAAGGTGTAAAATTGTAGTAATTTGAACCATGTAAGAG
V1119	CCAGACGTTTCCTCTCCCCACCGCGATCGAGCAAGGTTGCTCAAAATTTGTCTG
V0754	TTAAGATACATTGATGAGTTTGGACAAACCACAACTAGAATGC
V1118	GTGAATCGTGATTAAAAAGGACATtcggcatactcggtggcctcccc
V1117	GTCCAAACTCATCAATGTATCTTAacgttaactcgaatcgctatccaagc
V1062	TCGGcatactcggtggcctcc
V0335	TAAGATACATTGATGAGTTTGGACAAACCACAACTAGAATGCAGTGAAAAAAATG
V1029	ccgcaacctgtctctggtgatgGCCTCCTCCGAGGACG
V0981	GTTGGCGTTGACTTACTGAAAAG

V0957	CCGTGCATAATTCGATTTGATGCG
V1121	CCAGACGTTTCCTCTCCCCACCGCGATCGCGCTCTTCaGCTAGCCCACCCAATGTAC
V0754	TTAAGATACATTGATGAGTTTGGACAAACCACAACTAGAATGC
V1120	CGCATCAAATCGAATTATGCACGGtcggcatactcggtggcctccccac
V1060	CGCCACCACCTGTTCCTGTAGACATTGATGAGTTTGGACAAACCAC
V1059	CCGTCGTGGTCCTTATAGTCCATgtcacttggttgttcacgatcttg
V1167	ATGGACTATAAGGACCACGACGG
V0065	CCGAACAGGCCATTCTTCTTCTC
V0053	GAGAAGAAGAATGGCCTGTTCGG
V1061	CTACAGGAACAGGTGGTGGCG
V0621	GCAAGTTAAAATAAGGCTAGTCCGTTATCAACTTGAAAAAGTGGCACCGAGTCGGTGCATTTTTTTGGTGGC GGATGAGTAACAACATTC
V0622	CTTGCAAGTCGTCGTCGTCGTCTGGGAAGGGCGAATTCTGCAGATATC
V0623	CGGACTAGCCTTATTTTAACTTGCTATTTCTAGCTCTAAAACAGGTCTTCTCGAAGACCCAAGTTCGGAGGGT GCCCTCGTTGCTTATAT
V0624	CCAGACGACGACGACGACTTGCAAG
V0522	CAGTGCTGCCAGATTTTCGATAAAC
V0628	GGAGGCTCATTTCCTTAAACTCGTG
V1412	TCTAAAACGGGTCTTCGAGAAGACCTGAGTACAAAGGGCTCAAAATTTG
V0693	GCTAGAAATAGCAAGTTAAAATAAGGCTAGTCCGTTATCAACTTGAAAAAGTGGCACCGAGTCGGTGCTTTTT TTTTGATTCTGG
V0575	GCCGAAGCAAAGACAAGTTC
V0462	GTTTTACGGTGCACATCAATCTGAG
V1032	GTCCGTTATCAACTTGAAAAAGTGGCACCGAGTCGGTGCTTtTtttttGATTCTGGTTTaTTTTTGTTcTCTTTTGG AAG
V1034	TAGCCTTATTTTAACTTGCTATTTCTAGCTCTAAAACAGGTCTTCTCGAAGACCCGAGTACaaaGGGCTCAAAa TTTGCTTATATAG
V0465	GTATCCGTTGGCTAGTAGAAAAACTC
V0466	GCACTTCATTGCCATTCACAATAG
V1035	CCTTATTTTAACTTGCTATTTCTAGCTCTAAAACAGAGACGATCGTACGTCTCGAAaTTAtGTTGTCTACATATTT GCTTATATAGTTAG
V1036	CTAGTCCGTTATCAACTTGAAAAAGTGGCACCGAGTCGGTGCTTTtttttGGCCatAAACAATTTAAaTCG
V0695	GTTAAAATAAGGCTAGTCCGTTATCAACTTGAAAAAGTGGCACCGAGTCGGTGCTTTTTTTTTAGGGCGAATT CTGCAGATATCCATCAC
V0696	GTAGACCTTATTATCTTCGTAGATTTTGAAGGGCGAATTCCAGCAC
V0697	CAAAATCTACGAAGATAATAAGGTCTACTATTAC
V0698	TTGATAACGGACTAGCCTTATTTTAACTTGCTATTTCTAGCTCTAAAACAGGTCTTCTCGAAGACCCAAGTTGT AGGGCCGGGAAAGTGC
V0533	GCTCAAATTTTTGTGGGAGTTCCTTG
V0534	GGTTTTGGTAGAGAGTTGCTCTCTTATG
V1181	CTTGCTGTTTGGGGTCAATTCTTG
V1182	GTTATATATGGCCCATCAGGCTCG
V1184	ggaggccaccgagtatgCCGATTGCGGCAGATGATGCTGGAC
V1185	GTCCAAACTCATCAATGTATCTTAACTCGGATGCCATCAGCCATTCCCCG
V1186	GTACATTGGGTGGGCTAGCtGAAGAGCTTGCGGCAGATGATGCTGGACAGTTTC
V0540	GCTCTTCaGCTAGCCCACCCAATGTACCATAGTGCAAC
V0957	CCGTGCATAATTCGATTTGATGCG
941	GGGCGAATTCTGCAGATATCCATCAC
V1219	CTTGCAAGTCGTCGTCGTCGTCTGGGTTATATATGGCCCATCAGGCTCG
V1263	CAACTCGGATGCCATCAGCCAAGTTCG

V1267	GTTTcAGAGCTAtgctgGAAAcagcaTAGCAAGTTgAAATAAGGCTAGTCCGTTATCAAC
V1227	GTGATGGATATCTGCAGAATTCGCCCTAAGGAACACCTGTTCCCTCTGCGAG
451	GTACGCGTATCGATAAGCTTtaaGATACATTGATGAGTTTGG
611	CTGCGGCGATCGAAAGGCAAGGGCATTCAGC
V0308	CTTTCGATCGCCGCAGacgtcgtaaggctagttttgagaaggatcgcttgtctgggcaag
V0309	ATGTATCttaAAGCTTATCGATACGCGTACgctagccaaggtgctacgaaatccgttgtg
V0358	ctcaaaactagccttacgacgtGGCGATACTTGGATGCCCTGCGGCGATCGAAAGGCAAG
V0359	ATCCCCGGGCGAGCTCGcatatATCCGGGATGCGACTGCTCAATG
1068	atatgCGAGCTCGCCCGGGGATC
V1049	AtgctgTTTCcagcaTAGCTCTAAAACGATGGCGATACTTGGATGCCgacgttaaattg
V0357	acgtcgtaaggctagttttgagaaggatcgcttgtctgggcaag
V1048	TAGAGCTAtgctgGAAAcagcaTAGCAAGTTAAAATAAGGCTAGTC
V0897	gggccGCGACTCTAGATCATAATCAGCCATACCACAT
V1050	GATTATGATCTAGAGTCGCGGCCcCTATTACTTGTACAGCTCGTCCATG
V0359	ATCCCCGGGCGAGCTCGcatatATCCGGGATGCGACTGCTCAATG
V0897	gggccGCGACTCTAGATCATAATCAGCCATACCACAT
V1051	TCGTTTcAGAGCTAGAAATAGCAAGTTgAAATAAGGCTAGTCCGTTATCAAC
V1116	TGGCGATACTTGGATGCCgacgttaaattgaaaatagg
V1267	GTTTcAGAGCTAtgctgGAAAcagcaTAGCAAGTTgAAATAAGGCTAGTCCGTTATCAAC
V1268	GATGGCGATACTTGGATGCCgacgttaaattg
**Anneal Oligos for *kh-3* gRNA**	
V1141	aaacACCGAGTTTCGCTAAACTCC
V1142	ACTCGGAGTTTAGCGAAACTCGGT
**Anneal Oligos for *cd-1* gRNA**	
V1209	AAACCAACTCGGATGCCATCAGCC
V1212	ACTCGGCTGATGGCATCCGAGTTG
**Primers used for *cd-1* site Deep sequencing (amplicon added, marker as green)**	
V1217	ACACTCTTTCCCTACACGACGCTCTTCCGATCTCCGCTGATCACGTGCCTTCTCGG
V1218	GACTGGAGTTCAGACGTGTGCTCTTCCGATCTGCACTTGTTCCCACGCTGGCTTC
**Primers used for *Kh-3* site Deep sequencing (amplicon added, marker as green)**	
V1157	ACACTCTTTCCCTACACGACGCTCTTCCGATCTTGGTCATGATCAAGTGCAAACCG
V1158	GACTGGAGTTCAGACGTGTGCTCTTCCGATCTAAGGAAACGGAGTTGTACAGCG
**Primer used for NGS analysis in Hsu cells (*Kh-3* target site)**	
NGS218	ATGCGGCACACGCCATGGTT
NGS219	ACGAGTCTTCCTACCTCGATGTAGTTGTA
**Primers used to isogenize at the *cardinal* locus**	
V0917	GACCCTGATGAAACAAACACGC
V0918	CTTCAAGTGGCTGGCACCG

SUPPLEMENTARY DATA

Supplementary Data 1 - Genome editing quantification data.

Summary of the CRISPResso2 batch analysis on allele editing frequencies performed on deep sequencing data derived from *in vivo* and *in vitro* experiments. For each sample listed in tab 1 and tab 2 are specified: experimental details including names and ratio (%) of plasmid DNA transfected/injected and the experimental group; The raw CRISPResso allele frequency analysis output; The data extrapolated from the batch analysis to calculate allele editing and plotted in Fig1 and Supplementary Figure 2. The data as described is subdivided in the following file tabs:

1. **Cell line editing quantification**
2. **Embryo editing quantification**
3. **CRISPResso2 parameters:** Includes the running parameters used for all analysis on CRISPResso2 with one example representative of each batch experiment run.

Supplementary Data 2 - ***Culex quinquefasciatus*** **transgenesis counting data.**

Different injection conditions with G0 survival efficiencies were listed. Raw counting data of the G1 progeny phenotype indicating the cutting/ transgenesis in different conditions. *cd-/cd-* phenotype indicates the cutting and DsRed+ indicates the integration. All G0s were divided as male and female pools and crossed with *cd-/cd-*. Egg rafts from each male pool were scored together, while rafts of each female pool were divided and hatched singly in different batches, which gives more precise evaluation of germline rates. The data is subdivided in the following file tabs:

1. **Figure 2 - Table 1 Data:** The cutting/transgenesis efficiencies in conditions of injecting different HDR template variants (HR+Original gRNA; HR+"Loop"; HR+"Loop+Mutation") were recorded. The germline and overall efficiencies were calculated.
2. **Supplementary Table 1 Data:** The cutting/transgenesis efficiencies in other conditions by injecting HDR templates supplemented with Cas9 resources (Cas9 protein; Cas9 plasmid mixture; and both) were recorded. The germline and overall efficiencies were calculated.
3. **Statistical Analysis.** A Randomization Test for a Difference in Proportions was performed to evaluate differences between: 1) "Original" injection vs. "Loop" injection (per G0 germline); 2) "Original" injection vs. "Loop+mutation" injection (per G0 germline); 3) "Original" injection vs. "Loop" injection in overall scored G1s; and 4) "Original" injection vs. "Loop+mutation" injection in overall scored G1s.

Supplementary Data 3 - Cas9 line validation counting data.

Two experimental conditions of injecting *in-vitro*-transcribed *Kh3*-gRNA or *Kh3*-gRNA plasmid were listed. The embryos of the heterozygous Cas9 line were used for injection. The injection conditions and G0 situations were recorded. The Cas9 positive G0s were crossed with *kh-/kh-* mutants to evaluate gRNA activity in G1. The cutting efficiencies were calculated based on numbers of individuals giving *kh-* mutant phenotype in G1s. The data is subdivided in the following file tabs:

1. **Figure 3e Data:** The editing rate observed for either IVT-gRNA or plasmid is calculated.

Supplementary Data 4 - Effect of the gRNA scaffold with the loop modification on transgenesis efficiency.

Raw counting data of the G1 progeny phenotypic scoring indicating total females and males screened. All G0 crosses were performed in single-pairs between one injected individual and one wildtype (non-injected) animal. Number of recovered GFP positive (GFP+) and non-GFP (GFP-) individuals from the G1 are indicated. This allows us to precisely evaluate single-germline transgenesis rates in our experimental conditions. The data is subdivided in the following file tabs:

1. **Fig. 4 (w5 original)**: Trangenesis rates after injecting the w5-gRNA element carrying the "Original" gRNA scaffold version. Independent germlines that gave rise to transformants (GFP+) and total germlines analyzed as well as transformation rates are indicated.
2. **Fig. 4 (w5 loop)**: Trangenesis rates after injecting the w5-gRNA element carrying the "loop" gRNA scaffold modification. Independent germlines that gave rise to transformants (GFP+) and total germlines analyzed as well as transformation rates are indicated.
3. **Statistical Analysis.** Test for the Difference in G0 germline Proportions between w5 "Original" injection vs. w5 "Loop" injection, and Test for the Difference in G1 scored individuals Proportions between w5 "Original" injection vs. w5 "Loop" injection.

Supplementary Data 5 - Gene drive experiments with different gRNA scaffold variants in *Drosophila melanogaster*.

Raw counting data of the F2 progeny phenotypic scoring indicating females and males recovered. Red marker (DsRed+), green marker (GFP+), both fluorophores (both), no fluorescence (none), wild-type eye (w+), white-eye (w-), and mosaic eyes (mosaic) were scored in order to track Cas9 (red marker) and the gRNA (green marker) transgenes, as well as other outcomes of the cross. Transgene inheritance rates in the F2 progeny for each specific tube (marked as "F1 Cross" in the table) were calculated by combining data from males and females. Average inheritance for both markers and the standard deviation are calculated as well. The data is subdivided in the following file tabs:

1. **Fig. 5 (Original):** Inheritance of the *w5*-CopyCat targeting the *white* gene carrying the "Original" gRNA scaffold variant, driven by a transgene expressing Cas9 under the *vasa* promoter.
2. **Fig. 5 (Loop):** Inheritance of the *w5*-CopyCat carrying the "Loop" gRNA scaffold variant.
3. **Fig. 5 (Mutation):** Inheritance of the *w5*-CopyCat carrying the T>C "Mutation" gRNA scaffold variant.
4. **Fig. 5 (Loop+Mutation):** Inheritance of the *w5*-CopyCat carrying the gRNA scaffold variant with both the additional "Loop" as well as the T>C "Mutation".
5. **Summary:** This tab summarizes the total of flies counted in the four experimental conditions, and reports the average values for inheritance, cutting and conversion rates observed.
6. **St: Inheritance rates statistics:** Statistical analysis comparing different inheritance rates observed. The "Loop", "Mutation" and the "Loop+Mutation" conditions were compared to the "Original" condition.
7. **St: Cutting rates statistics:** Statistical analysis comparing different cutting rates comparison. The "Loop", "Mutation" and the "Loop+Mutation" conditions were compared to the "Original" condition.

8. **St: Conversion efficiency statistics:** Statistical analysis comparing different conversion efficiencies comparison. The “Loop”, “Mutation” and the “Loop+Mutation” conditions were compared to the “Original” condition.

SUPPLEMENTARY METHODS

Cloning details for the plasmid Constructs

Cas9 plasmids

1) **pVMG177 (*Cq-Actin5C*>Cas9_wHAs)**

The 5' regulatory sequence (~3.4kb upstream) and 3' terminator sequence (~0.4kb downstream) of *Cx. quin*-*Act5C* gene (Ref # CPIJ009808) were amplified from the genomic DNA of *Cx. quin*. CA strain and ligated with Cas9 protein (amplified from *Aste.* Cas9 plasmid) to build *Act5C*>Cas9 plasmid (**pVMG173**). The *Act5C*>Cas9 element then ligated with *Hr5IE1*>GFP fluorescent marker with Gibson Assembly. This component was later digested with BbsI enzyme and liagted with Cquin w4 HAs backbone (~1.0kb homology arms around the white-sgRNA-4 cutting site). The white gene (Ref # CPIJ005542) is amplified from *C. quin*. CA strain.

Primers used in this construct: 494, 495, V0915, V0889, V0888, V0914, V0700, V0638, V0882, V0883, V0325, V0884, V0885, 941, V0887, V0886.

2) **pVMG220** (***Cq-Rpl40*>Cas9_wHAs**)

The 5' regulatory sequence (~3.4kb upstream) and 3' terminator sequence (~0.9kb downstream) of *Rpl40* gene (Ref # CPIJ002413) were amplified from the genomic DNA of *C. quin*. CA strain and ligated with Cas9 protein (amplified from *Aste.* Cas9 plasmid) to build Rpl40>Cas9 plasmid (**pVMG193**). The Act5C>Cas9 element then ligated with *Hr5IE1*>GFP fluorescent marker with Gibson Assembly. This component was later ligated with *C. quin* w4 HAs backbone.

Primers used in this construct: V0834, V0832, V0963, V0964, V0325, V0884, V1044, V1040, V0512, V0408, V1068, V1069, V1000, V1070.

3) **pVMG235 (*Cq-nanos*>Cas9_wHAs)**

The 5' regulatory sequence (~3.3kb upstream) and 3' terminator sequence (~1.4kb downstream) of *nanos* gene (Ref # CPIJ011551) were amplified from the genomic DNA of *C., quin*. CA strain and ligated with Cas9 protein (amplified from *Aste.* Cas9 plasmid) to build *nanos*>Cas9 plasmid (**pVMG212**). The *nanos*>Cas9 element then ligated with Opie2>DsRed fluorescent marker with Gibson Assembly. This component was later ligated with *C. quin* w4 HAs backbone.

Primers used in this construct: V0660, V0678, V1009, V1011, V0325, V0884, V0982, V0656, V1010, V1119, V0754, V1118, V1117, V1068, V1062, V0335, V1029.

4) **pVMG236 (*Cq-vasa*>Cas9_wHAs)**

The 5' regulatory sequence (~2.4kb upstream) and 3' terminator sequence (~0.8kb downstream) of *vasa* gene (Ref # CPIJ009286) were amplified from the enomic DNA of *C. quin*. CA strain and ligated with Cas9 protein (amplified from *Aste.* Cas9 plasmid) to build *vasa*>Cas9 plasmid (**pVMG213**). The *vasa*>Cas9 element then ligated with Opie2>DsRed fluorescent marker with Gibson Assembly. This component was later ligated with *C. quin* w4 HAs backbone.

Primers used in this construct: V0957, V0981, V0325, V0884, V1013, V0540, V1014, V1012, V0957, V1121, V0754, V0335, V1029, V1062, V1028, V0754, V1120, V1117, V1068.

5) **pVMG225 (*IE1*>Cas9-P2A-DsRed)**

The 5’ regulatory sequence (~1.1kb upstream) and 3’ terminator sequence (~0.2kb downstream) of *Hr5IE1* gene were amplified from Baculovirus and ligated with Cas9 protein and T2A>DsRed using Gibson Assembling.

Primers used in this construct: V0887, V0886, V1060, V1059, 1167, V0065, V0053, V1061.

gRNA plasmids

1) **pVMG146 (*Cq*-U6:1>2XBbsI-gRNA)**

The promoter and terminator sequences of U6:1 (CPIJ039653) were amplified from *Cquin*. AL line, and ligated with a double-BbsI restriction site linker for later insertion of different gRNAs.

Primers used in this construct: V0621, V0622, V0623, V0624.

2) **pVMG147 (*Cq*-U6:2a>2XBbsI-gRNA)**

The promoter and terminator sequences of U6:2a (CPIJ039728) were amplified from *Cquin*. AL line, and ligated with a double-BbsI restriction site linker for later insertion of different gRNAs.

Primers used in this construct: V0522, V0628, V1412, V0693.

3) **pVMG217 (*Cq*-U6:2b>2XBbsI-gRNA)**

The promoter and terminator sequences of U6:2b (CPIJ039728) were amplified from *Cquin*. CA line, and ligated with a double-BbsI restriction site linker for later insertion of different gRNAs.

Primers used in this construct: V0575, V0462, V1032, V1034.

4) **pVMG218 (*Cq*-U6:4>2XBsmBI-gRNA)**

The promoter and terminator sequences of U6:4 (CPIJ039801) were amplified from *Cquin*. CA line, and ligated with a double-BsmBI restriction site linker for later insertion of different gRNAs.

Primers used in this construct: V0465, V0466, V1035, V1036.

5) **pVMG149(*Cq*-U6:6>2XBbsI-gRNA)**

The promoter and terminator sequences of U6:6 (CPIJ039596) were amplified from *Cquin*. AL line, and ligated with a double-BbsI restriction site linker for later insertion of different gRNAs.

Primers used in this construct: V0695, V0696, V0697, V0698.

6) **pVMG164 (*Cq*-U6:7>2XBbsI-gRNA)**

The promoter and terminator sequences of U6:7 (CPIJ040693) were amplified from *Cquin*. CA line, and ligated with a double-BbsI restriction site linker for later insertion of different gRNAs. This plasmid was synthesized by IDT-Geneblock based on the amplified sequence.

Primers used in this construct: V0533, V0534.

The *cd1*-sgRNA and *Kh3*-sgRNA were synthesized and later replaced the BbsI restriction site to build different U6-gRNA plasmids for experiments.

Anneal oligos for *cd1*: V1209 and V1212

Anneal oligos for *kh3*: V1141 and V1142

HDR templates for *Culex quinquefasciatus* transgenesis

1) **pVMG252 (*Cq-vasa*>Cas9_cdHAs_O)**

   The *vasa*>Cas9 element was amplified from **pVMG213** then ligated with Opie2>DsRed marker, and this transgene later inserted between two ~1.5 kb homology arms (HAs) matching the genomic sequences of the *cardinal* locus (CPIJ005949) abutting the *cd1*-gRNA target site. The U6:1>cd1-gRNA component was placed outside of the HAs.

   Primers used in this construct: V1181, V1182, V1185, V1184, V0754, V1062, V1186, V1120, V0540, V0957, 941, V1219, V0624, V1227.

2) **pVMG280 (*Cq-vasa*>Cas9_cdHAs_L)**

   The HAs and transgene are the same as **pVMG252**, the difference is the gRNA scaffold with a modified "Loop" structure.

   Primers used in this construct: V1181, V1182, V1185, V1184, V0754, V1062, V1186, V1120, V0540, V0957, V1263, V1264, V0624, V1227, 941, V1219.

3) **pVMG277 (*Cq-vasa*>Cas9_cdHAs_L+M)**

   The HAs and transgene are the same as **pVMG252**, the difference is the gRNA scaffold with a modified "Loop+Mutation" structure.

   Primers used in this construct: V1181, V1182, V1185, V1184, V0754, V1062, V1186, V1120, V0540, V0957, V1263, V1267, V0624, V1227, 941, V1219.

Copy-Cat constructs used in *Drosophila* experiments

1) **pVMG109 (CC-U6:3-*w5*-GFP-wHAs_O)**

   The *Dmel*-U6:3>w5 were synthesized by GeneBlock (IDT), and ligated with the 3XP3 driving EGFP fluorescent marker. These components were later inserted into the *drosophila white* gene at w5 cutting site with ~1.0kb homology arms.

   Primers used in this construct: 451, 611, V0308, V0309, V0358, V0359, 1068, V0357.

2) **pVMG221** (**CC-U6:3-*w5*-GFP-wHAs_L**)

   This construct was built based on the **pVMG109** with a "Loop" modification on its scaffold structure.

   Primers used in this construct: V0359, V1049, V1048, V0897, V1050, 1068.

3) **pVMG222** (**CC-U6:3-*w5*-GFP-wHAs_M**)

   This construct was built based on the **pVMG109** with a T>C "Mutation" modification on its scaffold structure.

   Primers used in this construct: V0359, V1049, V1048, V0897, V1050, 1068, V1051, V1116.

4) **pVMG274** (**CC-U6:3-*w5*-GFP-wHAs_L+M**)

This construct was built based on the **pVMG221** with both a C>T "Mutation" and "Loop" modifications on its scaffold structure.

Primers used in this construct: V1051, V1116, V1267, V1268.

Plasmid sequences and availability:

The plasmid sequences of the constructs generated in this manuscript are either deposited into the GenBank database or available from the authors upon request. GenBank accession numbers for the deposited plasmids are the following: pVMG0173_Cq-Actin5c-Cas9 (MW925696), pVMG0193_Cq-Rpl40-Cas9 (MW925697), pVMG0213_Cq-vasa-Cas9 (MW925698), pVMG0212_Cq-nanos-cas9 (MW925699), pVMG0146_Cq-U6-1_2xBbsI-gRNA (MW925700), pVMG0217_Cq-U6-2b_2xBbsI-gRNA (MW925701), pVMG0218_Cq-U6-4_2xBbsI-gRNA (MW925702), pVMG0149_Cq-U6-6_2xBbsI-gRNA (MW925703), pVMG0164_Cq-U6-7_2xBbsI-gRNA (MW925704), pVMG0252_Cq-vasa-Cas9_cdHAs_O (MW925705), pVMG0109_CC-U6-3-w5_GFP_wHAs_O (MW925706), pVMG0302_Cq-U6-1_2xBbsI_gRNA-Loop (MW925707), pVMG0303_Cq-U6-6_2xBbsI_gRNA-Loop (MW925708), pVMG0147_Cq_U6-2_2xBbsI-gRNA (MW925709). Selected plasmids have been deposited to the Addgene redistribution service, and are available for order by the community on the Addgene website (http://www.addgene.org/). Addgene identification numbers for the plasmids are as follows: pVMG0146_Cq-U6-1_2xBbsI-gRNA (169238), pVMG0149_Cq-U6-6_2xBbsI-gRNA (169323), pVMG0217_Cq-U6-2b_2xBbsI-gRNA (169339/), pVMG0173_Cq-Actin5c-Cas9 (169345), pVMG0193_Cq-Rpl40-Cas9 (169346), pVMG0213_Cq-vasa-Cas9 (169347), pVMG0212_Cq-nanos-cas9 (169348), pVMG0302_Cq-U6-1_2xBbsI_gRNA-Loop (169369), pVMG0303_Cq-U6-6_2xBbsI_gRNA-Loop (169370). Genbank and Addgene identification numbers are also available in Supplementary Fig. 1.

FASTA sequences of the gRNA scaffold variants used in this study.

>gRNAp-Original
GTTTTAGAGCTA-----GAAA-----TAGCAAGTTAAAATAAGGCTAGTCCGTTATCAACTTGAAAAAGTGGCACCGAGTCGGTGCTTTTTTT

>gRNA-Loop
GTTTTAGAGCTA**tgctg**GAAA**cagca**TAGCAAGTTAAAATAAGGCTAGTCCGTTATCAACTTGAAAAAGTGGCACCGAGTCGGTGCTTTTTTT

>gRNA-Mut
GTTT**c**AGAGCTA-----GAAA-----TAGCAAGTT**g**AAATAAGGCTAGTCCGTTATCAACTTGAAAAAGTGGCACCGAGTCGGTGCTTTTTTT

>gRNA-Loop+Mut
GTTT**c**AGAGCTA**tgctg**GAAA**cagca**TAGCAAGTT**g**AAATAAGGCTAGTCCGTTATCAACTTGAAAAAGTGGCACCGAGTCGGTGCTTTTTTT

Added loop sequences in red and mutation introduced in purple.
