# Supplementary material for: Optimized CRISPR tools and site-directed transgenesis towards gene drive development in Culex quinquefasciatus mosquitoes
Source: Nat Commun. 2021 May 20;12:2960. doi: 10.1038/s41467-021-23239-0 (PMC8137705; doi:10.1038/s41467-021-23239-0)
Supplement: Supplementary file 7 — Reporting Summary [file 41467_2021_23239_MOESM7_ESM.pdf]

nature research

Corresponding author(s): Valentino M. Gantz

Last updated by author(s): Apr 15, 2021

Reporting Summary

Nature Research wishes to improve the reproducibility of the work that we publish. This form provides structure for consistency and transparency in reporting. For further information on Nature Research policies, see our Editorial Policies and the Editorial Policy Checklist.

Statistics

For all statistical analyses, confirm that the following items are present in the figure legend, table legend, main text, or Methods section.

n/a	Confirmed	
[ ]	[x]	The exact sample size (n) for each experimental group/condition, given as a discrete number and unit of measurement
[ ]	[x]	A statement on whether measurements were taken from distinct samples or whether the same sample was measured repeatedly
[ ]	[x]	The statistical test(s) used AND whether they are one- or two-sided<br>*Only common tests should be described solely by name; describe more complex techniques in the Methods section.*
[ ]	[x]	A description of all covariates tested
[ ]	[x]	A description of any assumptions or corrections, such as tests of normality and adjustment for multiple comparisons
[ ]	[x]	A full description of the statistical parameters including central tendency (e.g. means) or other basic estimates (e.g. regression coefficient) AND variation (e.g. standard deviation) or associated estimates of uncertainty (e.g. confidence intervals)
[ ]	[x]	For null hypothesis testing, the test statistic (e.g. F, t, r) with confidence intervals, effect sizes, degrees of freedom and P value noted<br>*Give P values as exact values whenever suitable.*
[x]	[ ]	For Bayesian analysis, information on the choice of priors and Markov chain Monte Carlo settings
[x]	[ ]	For hierarchical and complex designs, identification of the appropriate level for tests and full reporting of outcomes
[x]	[ ]	Estimates of effect sizes (e.g. Cohen's d, Pearson's r), indicating how they were calculated

Our web collection on statistics for biologists contains articles on many of the points above.

Software and code

Policy information about availability of computer code

Data collection	No software was used for data collection.
Data analysis	Some of the collected data was analyzed using the CRISPResso online resource (https://crispresso.pinellolab.partners.org/) (CRISPResso2 version). CRISPResso parameters used in our data analysis are provided in the Supplementary Data 1 File. Graphical representation of the data and some statistical analysis was generated with GraphPad Prism 7. The additional software used for the statistical analysis was StatKey v. 2.1.1 .

For manuscripts utilizing custom algorithms or software that are central to the research but not yet described in published literature, software must be made available to editors and reviewers. We strongly encourage code deposition in a community repository (e.g. GitHub). See the Nature Research guidelines for submitting code & software for further information.

Data

Policy information about availability of data

All manuscripts must include a data availability statement. This statement should provide the following information, where applicable:

- Accession codes, unique identifiers, or web links for publicly available datasets
- A list of figures that have associated raw data
- A description of any restrictions on data availability

The plasmid sequences of the constructs generated in this manuscript are either deposited into the GenBank database or available from the authors upon request. GenBank accession numbers for the deposited plasmids are the following: pVMG0173_Cq-Actin5c-Cas9 (MW925696), pVMG0193_Cq-Rpl40-Cas9 (MW925697), pVMG0213_Cq-vasa-Cas9 (MW925698), pVMG0212_Cq-nanos-cas9 (MW925699), pVMG0146_Cq-U6-1_2xBbsI-gRNA (MW925700), pVMG0217_Cq-U6-2b_2xBbsI-gRNA (MW925701), pVMG0218_Cq-U6-4_2xBbsI-gRNA (MW925702), pVMG0149_Cq-U6-6_2xBbsI-gRNA (MW925703), pVMG0164_Cq-U6-7_2xBbsI-gRNA (MW925704), pVMG0252_Cq-vasa-Cas9_cdHAs_O (MW925705), pVMG0109_CC-U6-3-w5_GFP_wHAs_O (MW925706), pVMG0302_Cq-U6-1_2xBbsI_gRNA-Loop

(MW925707), pVMG0303_Cq-U6-6_2xBbsI_gRNA-Loop (MW925708), pVMG0147_Cq_U6-2_2xBbsI-gRNA (MW925709).Selected plasmids have been deposited to the Addgene redistribution service, and are available for order by the community on the Addgene website (http://www.addgene.org/). Addgene identification numbers for the plasmids are as follows: pVMG0146_Cq-U6-1_2xBbsI-gRNA (169238), pVMG0149_Cq-U6-6_2xBbsI-gRNA (169323), pVMG0217_Cq-U6-2b_2xBbsI-gRNA (169339/), pVMG0173_Cq-Actin5c-Cas9 (169345), pVMG0193_Cq-Rpl40-Cas9 (169346), pVMG0213_Cq-vasa-Cas9 (169347), pVMG0212_Cq-nanos-cas9 (169348), pVMG0302_Cq-U6-1_2xBbsI_gRNA-Loop (169369), pVMG0303_Cq-U6-6_2xBbsI_gRNA-Loop (169370). Genbank and Addgene identification numbers are also available in Supplementary Fig. 1. All source data are provided with this paper; they cover the raw phenotypical scoring data collected, which is reported in the Supplementary Data 2-5 files in Microsoft Excel format (.xlsx). All other data is available upon request from the authors.

Field-specific reporting

Please select the one below that is the best fit for your research. If you are not sure, read the appropriate sections before making your selection.

☒ Life sciences ☐ Behavioural & social sciences ☐ Ecological, evolutionary & environmental sciences

For a reference copy of the document with all sections, see nature.com/documents/nr-reporting-summary-flat.pdf

Life sciences study design

All studies must disclose on these points even when the disclosure is negative.

Sample size	For the cell work we maintained triplicates as our standard, which has allowed us to generate statistical comparisons between the different experimental conditions and identify significant differences. For in vivo analysis of cutting in embryos we only analyzed one sample; these experiments were meant to obtain a YES/NO answer on the activity of gRNAs and Cas9 constructs, and were mainly confirmatory of the information obtained from cells. Because of this reason we did not perform additional replicates and only one sample per condition was analyzed. For the transgenesis experiments we performed at least two injections per condition; the mosquito survival varied greatly, but we aimed at sampling at least 20 independent germlines to evaluate differences with statistical analysis. For Gene drive experiments, in our previous experience of similar analysis of gene drive effect using single fly pair crosses a number size of >8 is usually representative, describing the overall behavior; for each of our experimental condition we have collected between 21-37 different samples.
Data exclusions	All raw data provided was included in the figures. Fruit fly crosses with no progeny due to contamination or other causes, were removed from the analysis and are not reported in the raw data tables.
Replication	For the mosquito transgenesis experiments we performed two technical replicates. Not all replicates produced transgenesis, yet most produced evidence of cutting under the action of the Cas9/gRNA couple. Mosquito transgenesis experiments are technically challenging and notoriously difficult, with low survival rates and low transgenesis rates, and therefore, it is not unusual to perform injection rounds and not obtain trangenic animals. To further increase confidence in our claims we then proceeded to further support our observation with a similar experiment in Drosophila, a separate model organism.
Randomization	30 random larvae were picked for the molecular analysis performed in Fig. 1. F1 female flies were randomly collected from different F0 crosses to perform F1 crosses. No randomization practices are applicable to the remaining experiments.
Blinding	For all the mosquito/fly experiments performed we have analyzed fluorescence presence in the eyes of fruit flies or whole body for the mosquitoes. This type of scoring does not need the investigators to be blind as the evaluation of the phenotype is presence or absence of the fluorescent marker and there is no much room for interpretation that could be subjective.

Reporting for specific materials, systems and methods

We require information from authors about some types of materials, experimental systems and methods used in many studies. Here, indicate whether each material, system or method listed is relevant to your study. If you are not sure if a list item applies to your research, read the appropriate section before selecting a response.

Materials & experimental systems

n/a	Involved in the study	
☒	☐	Antibodies
☐	☒	Eukaryotic cell lines
☒	☐	Palaeontology and archaeology
☐	☒	Animals and other organisms
☒	☐	Human research participants
☒	☐	Clinical data
☒	☐	Dual use research of concern

Methods

n/a	Involved in the study	
☒	☐	ChIP-seq
☐	☒	Flow cytometry
☒	☐	MRI-based neuroimaging

Eukaryotic cell lines

Policy information about cell lines

Cell line source(s)	Culex quinquefasciatus Hsu cell line was kindly provided to the Perrimon Lab from Dr. Nelson Lau at Boston University, Boston MA. This line was originally generated by Hsu et al. (https://doi.org/10.1093/jmedent/7.6.703).
Authentication	The cells used were not autenticated.
Mycoplasma contamination	The cells were not tested for mycoplasma presence.
Commonly misidentified lines (See ICLAC register)	None were used

Animals and other organisms

Policy information about studies involving animals; ARRIVE guidelines recommended for reporting animal research

Laboratory animals	All Drosophila melonogaster transgenic animals were generated or assembled in Oregon-R genetic background. The Culex quinquefasciatus (California) strain was kindly provided by Anton Cornell (UC Davis), which was originally collected near the city of Merced, California in the 1950s. The Culex quinquefasciatus (Alabama) strain was kindly provided by Nannan Liu (Auburn University), which was collected from Huntsville, Alabama in 2002.
Wild animals	Study did not involve wild animals.
Field-collected samples	Study did not involve field-collected samples.
Ethics oversight	Drosophila melonogaster and Culex quinquefasciatus are invertebrate animals and are exempt from IACUC oversight.

Note that full information on the approval of the study protocol must also be provided in the manuscript.

Flow Cytometry

Plots

Confirm that:

- [x] The axis labels state the marker and fluorochrome used (e.g. CD4-FITC).
- [x] The axis scales are clearly visible. Include numbers along axes only for bottom left plot of group (a 'group' is an analysis of identical markers).
- [x] All plots are contour plots with outliers or pseudocolor plots.
- [x] A numerical value for number of cells or percentage (with statistics) is provided.

Methodology

Sample preparation	Cell sorting was performed in a Sony MA900 Multi-Application Cell Sorter (Department of Genetics, Harvard Medical School) using a 100µm sorting chip and average flow rate of 7000 events/second in semi-purity modality (~95 purity). Gates for cell singlets were defined based on forward and back scattering and a subordinate sorting gate was defined based on un-transfected control cells to sort GFP expressing cells. At the moment of sorting, stored cells were thawed quickly in a 30℃ water bath, washed twice in PBS, and resuspended in fresh culture media. For each sample, 5 x 10^5 GFP-positive cells were sorted directly in a 15ml falcon tube containing media, and refrigerated at 4℃ for the length of sorting. The sorted cells were pelleted and stored at -80℃ until genomic DNA extraction.
Instrument	Sony MA900 Multi-Application Cell Sorter (Department of Genetics, Harvard Medical School)
Software	Sony MA900 Sorter Software (the software comes with the instrument)
Cell population abundance	Cell purity after sorting was determined to be ~95% purity.
Gating strategy	Gates for cell singlets were defined based on forward and back scattering and a subordinate sorting gate was defined based on un-transfected control cells to sort GFP expressing cells

- [x] Tick this box to confirm that a figure exemplifying the gating strategy is provided in the Supplementary Information.
