# Supplementary material for: Optimized CRISPR tools and site-directed transgenesis towards gene drive development in Culex quinquefasciatus mosquitoes
Source: Nat Commun. 2021 May 20;12:2960. doi: 10.1038/s41467-021-23239-0 (PMC8137705; doi:10.1038/s41467-021-23239-0)
Supplement: Supplementary file 8 — Description of Additional Supplementary Files [file 41467_2021_23239_MOESM8_ESM.pdf]

Title: Supplementary Data 1 - Genome editing quantification data.

Description: Summary of the CRISPResso2 batch analysis on allele editing frequencies performed on deep sequencing data derived from in vivo and in vitro experiments. For each sample listed in tab 1 and tab 2 are specified: experimental details including names and ratio (%) of plasmid DNA transfected/injected and the experimental group; The raw CRISPResso allele frequency analysis output; The data extrapolated from the batch analysis to calculate allele editing and plotted in Fig1 and Supplementary Figure 2.

Title: Supplementary Data 2 - Culex quinquefasciatus transgenesis counting data.

Description: Different injection conditions with G0 survival efficiencies were listed. Raw counting data of the G1 progeny phenotype indicating the cutting/ transgenesis in different conditions. cd-/cd- phenotype indicates the cutting and DsRed+ indicates the integration. All G0s were divided as male and female pools and crossed with cd-/cd-. Egg rafts from each male pool were scored together, while rafts of each female pool were divided and hatched singly in different batches, which gives more precise evaluation of germline rates.

Title: Supplementary Data 3 - Cas9 line validation counting data.

Description: Two experimental conditions of injecting in-vitro-transcribed Kh3-gRNA or Kh3-gRNA plasmid were listed. The embryos of the heterozygous Cas9 line were used for injection. The injection conditions and G0 situations were recorded. The Cas9 positive G0s were crossed with kh-/kh- mutants to evaluate gRNA activity in G1. The cutting efficiencies were calculated based on numbers of individuals giving kh- mutant phenotype in G1s.

Title: Supplementary Data 4 - Effect of the gRNA scaffold with the loop modification on transgenesis efficiency.

Description: Raw counting data of the G1 progeny phenotypic scoring indicating total females and males screened. All G0 crosses were performed in single-pairs between one injected individual and one wildtype (non-injected) animal. Number of recovered GFP positive (GFP+) and non-GFP (GFP-) individuals from the G1 are indicated. This allows us to precisely evaluate single-germline transgenesis rates in our experimental conditions.

Title: Supplementary Data 5 - Gene drive experiments with different gRNA scaffold variants in Drosophila melanogaster.

Description: Raw counting data of the F2 progeny phenotypic scoring indicating females and males recovered. Red marker (DsRed+), green marker (GFP+), both fluorophores (both), no fluorescence (none), wild-type eye (w+), white-eye (w-), and mosaic eyes (mosaic) were scored in order to track

Cas9 (red marker) and the gRNA (green marker) transgenes, as well as other outcomes of the cross. Transgene inheritance rates in the F2 progeny for each specific tube (marked as “F1 Cross” in the table) were calculated by combining data from males and females. Average inheritance for both markers and the standard deviation are calculated as well.
